# Exploring Microalgae as a Novel Resource for Hepatocellular Carcinoma Therapy

**DOI:** 10.3390/molecules31061033

**Published:** 2026-03-19

**Authors:** Sik Yoon, Kok Keong Tan, Won Hoon Song, Chang Won Kim, Boon Huat Bay, Sae-Ock Oh

**Affiliations:** 1 Department of Anatomy, School of Medicine, Pusan National University, Yangsan 50612, Republic of Korea; 2 School of Biomedical Sciences, Li Ka Shing Faculty of Medicine, University of Hong Kong, Pokfulam, Hong Kong; kokkeong@hku.hk; 3 Department of Urology, School of Medicine, Pusan National University, Yangsan 50612, Republic of Korea; luchen99@hanmail.net; 4 Department of Radiology, School of Medicine, Pusan National University, Yangsan 50612, Republic of Korea; 5 Department of Anatomy, Yong Loo Lin School of Medicine, National University of Singapore, Singapore 117594, Singapore; antbaybh@nus.edu.sg

**Keywords:** hepatocellular carcinoma, microalgae-derived bioactive compounds, anticancer agents, chemoprevention, embolizing agents for transarterial chemoembolization, synergistic antitumor effects

## Abstract

Hepatocellular carcinoma (HCC) remains a major cause of cancer-related mortality in the world. Although there is an armamentarium of therapeutic options available for HCC therapy, current treatment modalities still face challenges, such as limited effectiveness and resistance to therapy due to inherent intratumoral heterogeneity. Hence, the development of novel therapeutics is an unmet need. Microalgae possess the ability to provide naturally derived compounds that are attractive for biomedical applications. The multifunctional nature of microalgae, with its unique combination of anticancer metabolites, oxygen-generating capability, and photosensitizing activity, make them a versatile platform for developing next-generation cancer therapeutics. In light of the above, this succinct narrative review highlights the potential biomedical applications of microalgae in cancer therapy, with a focus on HCC. Preclinical studies have shown the significant potential of microalgae as naturally occurring sources of chemopreventive and anticancer agents against HCC. Future directions include the use of biotechnology to enhance the production of microalgal-derived bioactive compounds and the formulation of biocompatible and biodegradable drug–microalgae embolic agents with prolonged release of anticancer drugs, thereby giving rise to synergistic antitumor effects, and their application for the delivery of immune checkpoint inhibitors for immunotherapy in HCC. Overall, microalgae hold considerable promise for advancing innovative therapeutic strategies against HCC.

## 1. Introduction

Hepatocellular carcinoma (HCC) has a high incidence and is one of the leading causes of cancer-related mortality globally. According to the GLOBOCAN 2020 database, HCC affected approximately 905,700 people worldwide in 2020 and is ranked as the third leading cause of cancer-related mortality [1,2]. The Barcelona Clinic of Liver Cancer (BCLC) algorithm is the most widely used staging system [3,4], with very early stage HCC classified as BCLC 0, early stage as BCLC A, intermediate stage as BCLC B, and advanced and terminal stages as BCLC C and BCLC D, respectively. Depending on the stage, distinct therapeutic strategies are recommended and instituted.

### 1.1. Current Treatment Modalities for HCC

The armamentarium of therapeutic options available for HCC therapy comprise surgery (liver resection and liver transplantation), ablation (thermal, radiofrequency or microwave), intra-arterial therapies, and systemic therapies [5].

(A) Surgery—Surgery may involve partial hepatectomy (partial resection of the liver) for HCC patients without cirrhosis, whereas liver transplantation is a potential option for patients with chronic liver disease and HCC [6].
(B) Ablation—Ablative modalities, which are minimally invasive procedures, include heat-based radiofrequency (RFA) and microwave ablation (MWA), and non-heat-based methods such as cryoablation and chemical ablation, with MWA as the most commonly used thermal ablative modality to destroy liver tumors [6].
(C) Intra-arterial therapies—Intra-arterial therapies involve the administration of vascular occlusive agents into the hepatic artery to cut off the blood supply of the hypervascular liver tumor tissues, thereby inducing hypoxia and necrosis, and include bland embolization (transarterial embolization or TAE), chemoembolization (transarterial chemoembolization or TACE) and radioembolization (transarterial radioembolization or TARE). TACE delivers chemotherapy directly into the liver tumor through the hepatic artery and then blocks the blood flow to the tumor. In contrast, TARE uses tiny radioactive beads instead of chemotherapy. These beads are delivered through the blood vessels directly into the tumor and emit radiation from inside [7,8].
(D) Systemic therapies—For patients with advanced disease, targeted therapy and immune checkpoint inhibitor (ICI) therapy have been introduced [9]. Several multikinase inhibitors, including sorafenib, lenvatinib, regorafenib, and cabozantinib—which target multiple tyrosine kinases as well as VEGFR2—have been employed as targeted therapies [10]. Moreover, ICIs such as nivolumab, pembrolizumab, and tremelimumab have demonstrated survival benefits in clinical trials [11].

### 1.2. Challenges to the Management of HCC

For patients with very early or early stage HCC (BCLC 0/A), the primary treatment strategies include surgery (liver resection and transplantation) and local ablation such as RFA and MWA. However, even after curative resection, recurrence rates can be as high as 60–80% within five years [12], giving rise to subsequent treatment challenges and influencing long-term survival outcomes. Liver transplantation, though ideal for selected patients, encounters difficulties such as organ shortage, risk of post-transplant tumor recurrence, and immunosuppressive complications [13]. Ablative therapies (e.g., RFA and MWA) are minimally invasive and effective in small tumors; however, they are less effective for tumors larger than 3 cm, or those located near major vessels due to the heat-sink effect, and have higher local recurrence compared to surgery [14].

Intermediate stage HCC (BCLC B) is a heterogeneous group, historically managed with TACE. However, recent evidence suggests that a personalized approach is necessary due to varied tumor burden and liver function among patients. Current treatment options for BCLC B also include TACE, TARE, combination therapies, and surgical resection. Patients under this classification are a heterogeneous group in terms of tumor number, size, and liver functional reserve. The efficacy of intra-arterial therapies such as TAE and TACE is limited by tumor revascularization, incomplete necrosis, and heterogeneous responses due to variable vascular anatomy [15].

For advanced HCC (BCLC C), systemic therapy is the standard of care. The development of novel agents has substantially shifted treatment paradigms in recent years. Key systemic therapies include ICIs and tyrosine kinase inhibitors (TKIs). Multikinase inhibitors have improved overall survival; however, issues such as primary and acquired drug resistance, tumor escape via alternative signaling pathways, and significant adverse effects, including hypertension, hand–foot skin reactions, and diarrhea, continue to limit their efficacy [16]. ICIs have significantly prolonged survival in several other types of cancer, yet their effectiveness in HCC remains limited [17,18]. This limitation is largely due to the relatively low tumor mutational burden in HCC, which results in restricted neoantigen expression, thereby reducing immune recognition [19]. Additionally, chronic inflammation, immune-tolerant liver microenvironment, and persistent antigenic stimulation lead to CD8^+^ T-cell exhaustion and multiple forms of immune suppression [19]. These factors hinder robust and sustained antitumor immunity.

Given the high degree of intratumoral heterogeneity in HCC, monotherapy often results in therapeutic resistance. To overcome these barriers, various combination strategies—such as integrating local and targeted therapies, immune and targeted therapies, or local and immune therapies—are being explored [19,20,21]. Nonetheless, the lack of robust predictive biomarkers, early detection methods, and personalized therapeutic approaches underscores the urgent need for novel therapeutic modalities and precision medicine frameworks in HCC management.

This concise narrative review highlights the potential biomedical applications of microalgae in cancer therapy, with a focus on HCC.

## 2. Microalgae as Sources of Bioactive Compounds for Biomedical Applications

Microalgae are unicellular photosynthetic eukaryotes, whereas cyanobacteria are prokaryotic “blue-green algae” that share comparable physiological and ecological characteristics, including overlapping metabolite profiles [22,23,24]. For the purposes of this review, microalgae and cyanobacteria are therefore considered collectively, and the term “microalgae” is used broadly to encompass cyanobacteria where relevant. The extensive diversity of microalgal species constitutes a rich reservoir of bioactive compounds with potential applications across numerous biotechnological and industrial sectors, including food, health, energy, biomaterials, animal husbandry, aquaculture, cosmetics, and environmental management [25,26,27,28,29,30,31].

**Figure 1:**
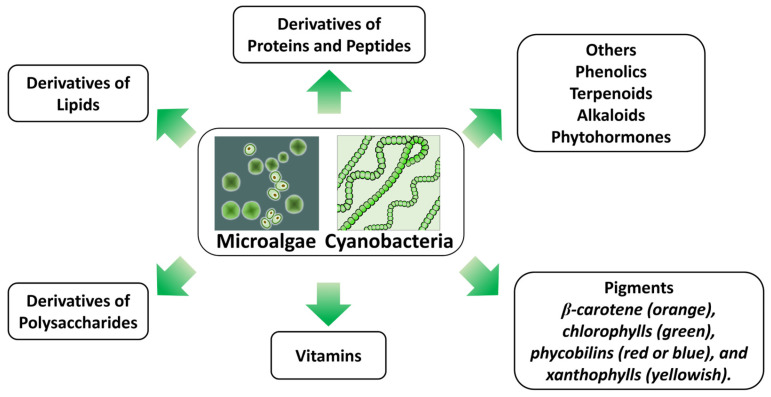
Bioactive compounds derived from microalgae and cyanobacteria.

Bioactive compounds derived from microalgae include proteins, peptides, lipids, polysaccharides, vitamins, pigments, terpenoids, alkaloids, and other specialized metabolites (Figure 1). Microalgae also synthesize phytohormones such as auxin, abscisic acid, cytokinin and polyamines, which regulate essential physiological processes in both terrestrial and aquatic organisms [32].

Collectively, these metabolites constitute a diverse pool of physiologically active molecules with antioxidant, antimicrobial, anti-enzymatic, antibiotic, anti-inflammatory, photoprotective, anti-aging, and hypocholesterolemic properties [33]. Microalgal biosynthesis is governed by complex metabolic pathways and is shaped by numerous biotic and abiotic environmental factors [34], positioning microalgae as promising and sustainable feedstocks for the development of high-value bioactive products [35].

Microalgae have therefore garnered considerable interest as natural sources for the production of bioactive compounds. Although many commercial bioactive molecules are obtained through chemical synthesis, synthetic analogs often differ from their natural counterparts in structural complexity and stereochemical configuration, which can influence safety and biological activity [36]. For instance, synthetic astaxanthin contains a mixture of stereoisomers, whereas natural astaxanthin from microalgae consists predominantly of the 3S,3′S enantiomer [37]. Astaxanthin derived from *Haematococcus pluvialis* exhibits markedly stronger antioxidant activity than synthetic astaxanthin [37], and the natural 3S,3′S form provides superior pigmentation in rainbow trout, making it a preferred additive in aquaculture feed [38].

Microalgal metabolites such as carotenoids and polyunsaturated fatty acids (PUFAs) are already widely utilized in the food, cosmetic, and pharmaceutical sectors [39]. These bioactive products demonstrate diverse biological activities, including antioxidant, anti-inflammatory, antiviral, antibacterial, antifungal, and antitumor effects [40]. Despite these promising attributes, the full therapeutic and industrial potential of microalgal metabolites remains underexplored.

Nevertheless, microalgae offer several advantages over terrestrial plants and synthetic production systems, including rapid growth rates, scalability, the use of non-arable land, and—in carefully selected non-toxic strains—robust biosynthetic capacity for generating naturally derived compounds suited for biomedical, nutraceutical, and cosmeceutical applications [39,41]. These advantages underscore the practicality and importance of developing microalgae as sustainable platforms for the large-scale production of high-value bioactive metabolites.

## 3. Microalgae-Derived Anticancer Agents

Microalgae are increasingly recognized as valuable sources of pharmacologically active metabolites with anticancer potential. Their diverse metabolic profiles—including pigments, polysaccharides, proteins, terpenoids, PUFAs, vitamins, and phenolic compounds—enable the production of numerous bioactive molecules with therapeutic relevance [42]. These metabolites exert anticancer effects through various mechanisms, such as impeding cell proliferation, inducing apoptosis, regulating cell-cycle progression, and suppressing cancer cell invasion [43,44,45]. In addition, beyond naturally occurring free fatty acids, recent research has highlighted the potent anticancer activity of their chemical derivatives, specifically fatty acid potassium salts and fatty acid lithium salts. These water-soluble salts, often derived from microbial sources like *Nannochloropsis salina* or oleaginous fungi, have demonstrated significant lethality against cancer cell lines—including those of the breast and prostate—by suppressing proliferation and migration [46,47]. Such findings suggest that the conversion of microalgae-derived lipids into alkali salts could be an effective strategy to enhance their bioavailability and synergistic antitumor potential in cancer therapy. Extracts from *Chlorella*, *Spirulina* (also known as *Arthrospira*), and multiple cyanobacterial species have demonstrated selective cytotoxicity against cancer cells by enhancing apoptosis, producing cell-cycle arrest, impairing mitochondrial function, and attenuating tumor-promoting inflammation [42,48].

Key microalgal metabolites—including c-phycocyanin, fucoidan-like sulfated polysaccharides, carotenoids, phlorotannins, flavonoids, and terpenoids—have been shown to inhibit tumor cell proliferation, reduce metastasis through the modulation of matrix metalloproteinases, and enhance endogenous antioxidant defenses that counteract oxidative stress associated with cancer progression [49,50,51,52,53]. Beyond extract-based compounds, whole cells from microalgae and related microorganisms, such as *Chlorella*, *Chlamydomonas reinhardtii*, *Shewanella algae*, and *Spirulina*, have exhibited intrinsic antitumor activity. These effects arise from cytotoxic metabolite production, the induction of mitochondrial-mediated apoptosis, and immune–stimulatory properties that support host antitumor responses [42,48,53,54,55,56,57,58,59,60,61]. Furthermore, the unique photosynthetic capability of microalgae has enabled therapeutic functions not seen in conventional anticancer agents. Upon light exposure, microalgae generate oxygen that can alleviate tumor hypoxia and synergistically enhance treatments such as radiotherapy and photodynamic therapy (PDT), while chlorophylls and related pigments serve as endogenous photosensitizers, producing reactive oxygen species (ROS) to mediate tumor ablation [42,62,63,64,65,66,67,68,69,70].

**Figure 2:**
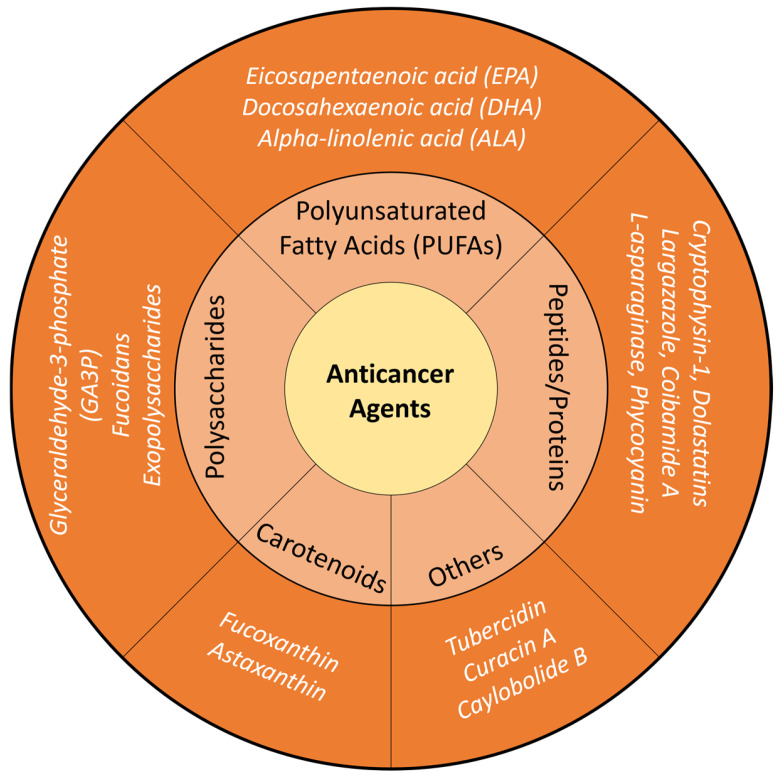
Microalgal-derived anticancer agents.

The multifunctional nature of microalgae—including their production of anticancer metabolites, oxygen-generating capacity, and photosensitizing activity—positions them as sustainable, versatile, and highly adaptable platforms for the development of next-generation cancer therapeutics [44,45]. A schematic of microalgae-derived anticancer agents is shown in Figure 2.

The key anticancer compounds derived from microalgae, including carotenoids, PUFAs, polysaccharides, proteins, peptides, and other metabolites, together with their source organisms, primary anticancer mechanisms, and the most relevant cancer models studied, are summarized in Table 1.

## 4. Microalgae-Based Anticancer Agents for Targeting HCC

### 4.1. In Vitro Studies

#### 4.1.1. Microalgae-Derived Bioactive Compounds

##### Carotenoids

Carotenoids isolated from freshwater microalgae *Monoraphidium* sp. and *Scenedesmus obliquus* showed growth-inhibitory effects in Huh7 HCC cells [117]. The IC_50_ values (half maximal inhibitory concentration) of microalgae carotenoid fractions on Huh7 cells were observed to be 77.38 μg mL^−1^ and 1481.72 μg mL^−1^ for *Monoraphidium* sp. and *Scenedesmus obliquus*, respectively, showing that carotenoids extracted from *Monoraphidium* sp. had a much higher antiproliferative effect. The same investigators noted that the *Monoraphidium* sp. carotenoid extract was rich in astaxanthin and exhibited substantially higher free radical scavenging activity as compared to the *Scenedesmus obliquus* extract (55.7% vs. 25.9%), which possessed β-carotene and a lower astaxanthin content. The above study suggests that carotenoids, especially astaxanthin, may appear as a potential agent to be explored for the treatment of HCC.

A fucoxanthin-rich fraction from *Chaetoceros calcitrans* extract has been reported to induce cytotoxicity in HepG2 cells more effectively than the crude extract in both a dose-dependent and time-dependent manner, with an IC_50_ of 18.89 μg mL^−1^ compared to 87.5 μg mL^−1^, respectively [72]. The effect was observed to be mediated through multiple signaling pathways, including oxidative stress, that regulate apoptotic cell death.

Another study demonstrated that two compounds, loliolide and epi-loliolide, isolated from the dichloromethane extract of the unicellular flagellated microalga, *Tisochrysis lutea*, exhibited cytotoxic effects against HepG2 cells, with epi-loliolide having a higher antitumor activity [118]. Both compounds which are carotenoid-derived metabolites were also observed to induce less toxicity towards non-tumoral murine stromal S17 cells. Although the mechanism is hitherto unknown, stereoisomers of carotenoid metabolites, such as loliolide, have a wide spectrum of biological properties.

##### Proteins

A research team employing a new bioalgorithm using proteomic data (previously generated by the group) from *Microchloropsis gaditana* (formerly known as *Nannochloropsis gaditana*) to compare with an existing patent database identified and selected the UCA01 protein for further analysis [119]. The UCA01 protein belongs to the multifunctional prohibitin family, which is known to have antiproliferative effects. The recombinant UCA01 protein was shown to have a significant inhibitory effect on the growth of HepG2 cells and Caco-2 (colon adenocarcinoma) cells, but not on control EA.hy926 endothelial cells.

C-phycocyanin, a water-soluble pigment–protein complex from *S. platensis*, has been shown to induce apoptosis in doxorubicin-resistant HepG2 cells [120]. Doxorubicin (DOX) is a chemotherapeutic drug and the same study revealed that the induction of apoptosis in DOX-resistant HepG2 cells was mediated by an alteration in the mitochondrial membrane potential with the downregulation of anti-apoptotic protein Bcl2 and the upregulation of pro-apoptotic Bax protein. Similarly, phycocyanin extracted from *Microcystis aeruginosa* induced apoptosis in HepG2 cells subjected to phycocyanin-mediated PDT [121]. In this study, Microcystis–phycocyanin PDT was observed to effectively inhibit HepG2 cell proliferation causing mitochondrial damage, culminating in apoptosis through a mitochondria-dependent pathway. In addition, c-phycocyanin from *S. platensis* significantly reduced the invasive potential of HepG2 cells by inhibiting MMP-2 and MMP-9 activity and expression, accompanied by the downregulation of TIMP-2, which would have implications for the suppression of HCC spread [122].

##### Polysaccharide

A novel high-molecular-weight *Cyanobacterium aponinum* polysaccharide (CAP) was isolated and purified from crude extracts using column chromatography [123]. Further investigations revealed that CAP exhibited significant growth-inhibitory effects against HepG2 cells but had no effect on controlling human fetal hepatocyte LO2 cells. Compared to the negative control, all treatment groups with different concentrations of CAP showed significant differences in the inhibition rates of HepG2 cells, highlighting its potential as a bioactive antitumor agent.

Furthermore, polysaccharides from *S. platensis* exhibited cytotoxic and pro-apoptotic effects against HepG2 cells and served as efficient biostimulants for the synthesis of silver nanoparticles (AgNPs), which further enhanced anticancer activity [124]. In this study, AgNPs were synthesized with soluble polysaccharides obtained from *Spirulina platensis* as reducing and stabilizing agents. The IC_50_ for the green synthesized biogenic AgNPs was lower than that of the soluble polysaccharides (24.5 versus 65.4 µg mL^−1^, respectively). Hence, green synthesized AgNPs could be further explored as a potential treatment strategy for HCC.

##### Polyketides

Amphifinol 22, belonging to the family of bioactive polyketides, isolated by the bioassay-guided fractionation of dinoflagellate *Amphidinium carterae* methanol crude extracts, was observed to decrease the cell viability of HepG2 cells [125]. In that study, amphifinol 22 was shown to exert cytotoxicity in A549 non-small cell lung cancer cells (IC_50_ 8 µM), A2058 melanoma cells (IC_50_ 16.4 µM), HepG2 cells (IC_50_ 6.8 µM), MCF7 breast cancer cells (IC_50_ 16.8 µM) and Miapaca-2 pancreatic cancer cells (IC_50_ 8.6 µM), with HepG2 cells having the lowest IC_50_ among the cell lines tested.

#### 4.1.2. Microalgae-Derived Crude Extracts

Methanol extracts from cyanobacteria *Jaaginema* sp. strains have been shown to exhibit potent cytotoxic activities against Huh7 and HepG2 cells, accompanied by cellular features suggestive of oxidative stress [126]. Methanol extracts of *Chlorella vulgaris* supplemented with thiamine have also been reported to exhibit a higher inhibition of cell growth of HepG2 cells than just the microalgae extract alone at all concentrations used [127]. In that study, the same trend was similarly observed in HCT-116 colorectal cancer cells, Hela cervical cancer cells and PC-3 prostate cancer cells treated with the *Chlorella vulgaris* extract. Furthermore, the antioxidant activity in extracts of thiamine-supplemented *Chlorella vulgaris* also showed higher antioxidant activity measured by the DPPH (1,1-Diphenyl-2-picryl-hydrazyl) antioxidant assay as compared with other vitamin supplementations of riboflavin, ascorbic acid and pyridoxal.

Another study demonstrated that ethanol extracts of microalga *Chlorella* species, but not cyanobacterium *Spirulina* species, could significantly decrease the cell viability of Huh7 cells [110]. However, *Spirulina platensis* had previously been reported to induce apoptosis and inhibit cell proliferation, associated with an increase in Bax/Bcl-2 ratio in HepG2 cells [128]. Wu et al. reported that aqueous extracts of both *Spirulina* and *Chlorella* exhibited antiproliferative effects in HepG2 cells, with *Spirulina* showing a superior effect [129].

Additionally, the F50 extract of the thermotolerant microalga *Coelastrella* sp., prepared using a methanol/dichloromethane mix, was observed to reduce the number and size of spheres formed by Huh-7 cells and N1-S1 rat hepatoma cells in the sphere formation assay [130]. *Coelastrella* F50 extract was also observed in this study to suppress self-renewal capability and asymmetric division. Additionally, the same study revealed a concomitant inhibition of cancer stem cell (CSC) markers CD133 and ABCG2 in a dose-dependent and time-dependent manner.

These findings underscore the potential of microalgae-derived compounds as natural therapeutic agents for HCC treatment.

### 4.2. In Vivo Studies

In vivo studies have also demonstrated that microalgae and cyanobacteria possess significant chemopreventive, antioxidative and antineoplastic activities in HCC (as summarized in Table 2).

**Figure 3:**
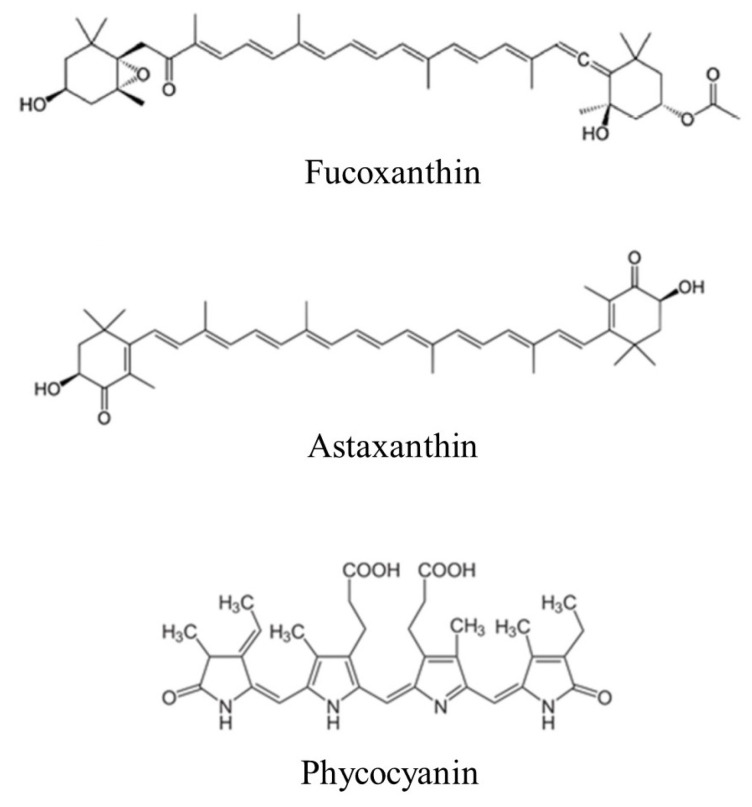
Potential lead compounds derived from microalgae for further exploration as anticancer agents. Chemical structures of the bioactive compounds are individually reproduced (in truncated form) from Figure 1 of Barkia et al. [136].

From the above literature survey, it would appear that phycocyanin has emerged as a very promising microalgae-derived lead compound for development as an HCC therapeutic. Phycocyanin exhibits intrinsic antitumor activity and is a potent photosensitizer for PDT, as supported by relatively robust preclinical evidence, including both in vitro and in vivo studies (see also Figure 3). Other potential candidates include fucoxanthin and astaxanthin, although additional in vivo investigations are required assess treatment efficacy and safety.

Beyond their direct intrinsic cytotoxic effects on cancer cells, microalgae-derived bioactive molecules have been reported to possess significant potential for applications in HCC therapy, such as:

(A) Embolization agent for TACE in HCC therapy

Recently, Wang et al. [137] developed a novel embolic agent by incorporating the chemotherapeutic drug, DOX, into *Spirulina* (*SP*) to formulate a biocompatible and biodegradable drug–microalgae embolization agent (DOX-SP), with high-performance embolization and sustained release of the anticancer drug, thereby giving rise to synergistic antitumor effects. An orthotopic murine xenograft model was first established by 5 × 10^6^ N1S1 cells into the left lateral lobe of the rat’s liver. Subsequently, DOX-SP was administered through a catheter placed in the left hepatic artery of tumor-bearing rats. The antitumor efficacy of DOX-SP on the rat N1S1 HCC was evident, as demonstrated by the effective suppression of tumor growth with significant reduction in tumor size, and causing minimal adverse effects.

(B) Drug delivery system

A group of investigators have recently developed a novel drug delivery system for a PD-1 inhibitor (Camrelizumab, an immunotherapy drug) by combining the protective and adhesive properties of polydopamine (PDA) with the biocompatibility and drug-loading capacity of *Chlorella vulgaris* microalgae (PDA-CV@PI) [138]. The subcutaneous mouse xenograft model was established by injecting B6-hPD1 mice with murine Hepa1–6 hepatoma cells (1 × 10^6^ cells) subcutaneously on the right hind limb, whereas the orthotopic model was developed by injecting 50 µL of Hepa1–6 cytosol (2 × 10^7^ mL^−1^) into the exposed mouse liver. PDA-CV@PI was administered to the tumor-bearing mice by oral gavage. The authors observed that PDA-CV@PI used in combination with irreversible electroporation (IRE or nanoknife ablation, a physical ablation method) significantly improved therapeutic efficacy with a concomitant reduction in PI (Camrelizumab) side effects. Hence, IRE and PDA-CV@PI could potentially be an effective strategy for augmenting the overall anticancer immune response.

## 5. Future Perspectives

**Figure 4:**
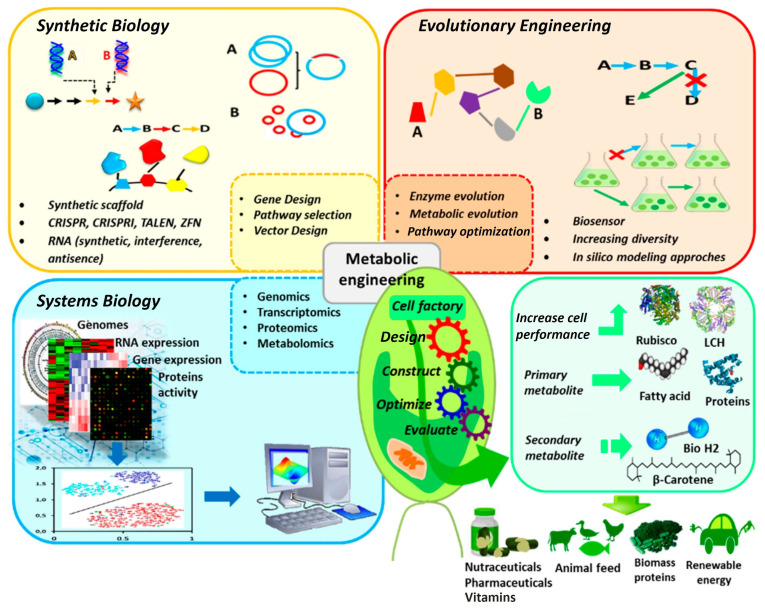
Genetic technology applied to microalgae to optimize production of target metabolites. Reprinted with slight modifications to the figure from Grama et al. [142].

For microalgal products to be feasible and sustainable for biomedical applications, a reliable and consistent supply of microalgal biomass is essential. The rapid generation times of microalgae and cyanobacteria have enabled efficient large-scale cultivation in photobioreactors, making them a renewable and still underexploited resource for producing pharmaceuticals and other high-value bioactive agents [31,139,140,141]. Ensuring compliance with Good Manufacturing Practice (GMP) standards is also critical for the safe and reproducible production of microalgal-derived compounds. Advances in microalgal biotechnology, particularly genetic engineering and metabolic optimization strategies, have opened new opportunities to improve both biomass productivity and metabolite yield. As schematically summarized in Figure 4, these approaches include genome editing technologies such as CRISPR-based systems, the overexpression or suppression of key metabolic pathway enzymes, and systems biology-guided pathway redesign [142]. Genome editing tools enable precise modification of endogenous genes to redirect metabolic flux toward desired products, while the overexpression of rate-limiting enzymes or transcriptional regulators enhances pathway efficiency. In parallel, systems biology-guided metabolic engineering integrates transcriptomic, proteomic, and metabolomic information to identify optimal intervention points. Together, these strategies, as highlighted by Grama et al., allow microalgal cells to function as engineered “cell factories,” leading to the increased accumulation of primary metabolites (e.g., lipids and proteins) and secondary metabolites (e.g., carotenoids and bioactive compounds), as conceptually depicted in Figure 4 [142].

Nonetheless, several challenges must be addressed to support large-scale deployment. Environmental risks—such as impacts on water quality, climate, and local biodiversity—require careful assessment and mitigation strategies [140]. Continued improvements in cultivation systems, process control, and regulatory frameworks will be essential to fully realize the biomedical potential of microalgal platforms.

From a future-oriented perspective, adaptive laboratory evolution (ALE) is emerging as a powerful strategy to boost the yields of microalgae-derived anticancer metabolites without requiring transgenic modification [143,144]. By subjecting microalgal cultures to prolonged selective stresses, ALE facilitates the accumulation of beneficial mutations that concurrently enhance stress tolerance and redirect metabolic flux toward the increased biosynthesis of target compounds. Indeed, evolved ALE strains have demonstrated a markedly higher production of potent bioactive agents (e.g., astaxanthin, fucoxanthin, phycocyanin, and DHA) known for their anticancer activity, achieved via multi-gene metabolic reprogramming that maximizes flux toward these metabolites [143,144].

For example, in a study on the diatom *Phaeodactylum tricornutum*, ALE using food waste-derived carbon sources improved the strain utilization of heterogeneous substrates, thereby increasing biomass and levels of PUFAs such as EPA and DHA, which are known for their anticancer potential [145]. Likewise, long-term high-temperature ALE in *Schizochytrium* sp. yielded a thermotolerant mutant (ALE70) capable of maintaining stable growth and producing more than four times the DHA yield of the wild type under 34.5 °C conditions—an advantage for cost-effective industrial fermentation [146]. In another case, a composite ALE strategy involving acid stress and oxygen tension in *Aurantiochytrium* sp. led to a 171% increase in DHA yield and 2.4-fold total lipid production, correlated with the widespread upregulation of genes in the polyketide synthase pathway and tricarboxylic acid cycle, as revealed by transcriptome analysis [147]. A distinctive example includes a self-flocculating strain of *Desertifilum* sp., which evolved CO_2_ tolerance up to 15% through gradient ALE. The evolved strain demonstrated enhanced nutrient removal from wastewater and significantly increased phycocyanin production—an antioxidant pigment with documented anticancer activity—under high-density cultivation [148].

Importantly, the anticancer efficacy of bioactive compounds derived from evolved strains has been demonstrated in specific cases, such as fucoxanthin-enriched extracts from ALE-treated microalgae [72]. For other microalgal and cyanobacterial metabolites, including phycobiliproteins, carotenoids, and PUFAs, ALE has been shown to enhance cellular productivity and metabolic performance, while the preservation of their established anticancer bioactivity represents an important research gap to be addressed in future studies [149,150].

Integrating ALE with comprehensive omics analyses can elucidate the genetic and metabolic adaptations underlying such improvements, offering insights to guide rational metabolic engineering or further tailored evolution strategies [151]. Moreover, the advent of automated, high-throughput ALE platforms combined with machine learning-guided experimental design is expected to accelerate the development of custom high-yield microalgal strains by enabling parallel evolution trials and efficient optimization of cultivation conditions [144].

Regarding the various treatment modalities of HCC therapy, TACE is a recommended treatment option for intermediate stage disease. Of the two forms of TACE, drug-eluting bead TACE (DEB-TACE) has advantages over conventional TACE (cTACE) because cTACE is characterized by inconsistent embolization and a rapid drug release that can result in an augmented risk of systemic toxicity [137]. However, a metanalysis showed similar safety profiles for patients treated with cTACE and DEB-TACE, but it also showed that DEB-TACE had a superior efficacy [152]. Yet, a separate meta-analysis performed earlier (with patients derived from four randomized and eight observational studies) revealed the lack of superiority of DEB-TACE over cTACE with regard to tumor response and survival [153]. It is exciting that Wang et al., as mentioned earlier, have developed an embolization agent DOX-SP for use in TACE [137].

Hitherto, embolizing agents for intra-arterial therapy include gelatin sponge particles, iodized oil (lipiodol), degradable starch microspheres for temporary arterial occlusion, and polyvinyl alcohol for permanent occlusion [154]. However, gelatin sponges and microparticles, which are manufactured from gelatin via partial hydrolysis of collagen, contain animal-based porcine or bovine gelatin (for example, commercially available Gelfoam^R^ and EGgel are produced from pig skin gelatin, while Spongel is derived from cattle hide and bones), which may have religious connotations. Hence, it is also timely to consider alternatives to animal-based products such as microalgae and microalgae-derived compounds as embolizing agents for patients who are undergoing cTACE for HCC.

Immunotherapy, which includes the use of dual ICIs such as atezolizumab–bevacizumab and durvalumab–tremelimumab, is a recommended treatment for advanced HCC [155]. The recent development of a potent drug delivery system for an ICI, based on an oral composite platform, represents a significant advancement in HCC treatment, which, when used in combination with IRE as previously stated, offers a novel strategy for HCC therapy [138]. Using microalgae as natural drug delivery systems for HCC, and capitalizing on their inherent tumor targeting ability to enhance drug accumulation with the minimization of side effects, represents a promising and innovative therapeutic strategy.

Although synthetic analogs may exhibit certain disadvantages relative to the natural compounds, such as reduced biological activity, they could also offer a new avenue for translating microalgae bioactive molecules into HCC therapy. For instance, Cai et al. [156] have designed and synthesized a novel apratoxin analog S10, which was observed to have a potent antiproliferative effect in Huh7 cells by the downregulation of receptor tyrosine kinases (RTKs), with a correspondingly much lower IC_50_ compared to RTK inhibitors, Sunitinib, Erlotinib and cabozantinib. However, further in vivo studies are required to fully evaluate its bioavailability, pharmokinetics, therapeutic efficacy and safety profile [157].

## 6. Conclusions

Microalgae hold great promise as naturally occurring sources of anticancer agents against HCC and for chemoprevention of this dreaded disease. However, it is still early days, as only preclinical studies on its antitumor efficacy and safety have been conducted thus far. To our knowledge, there have been no clinical trials conducted on microalgae-based therapeutics for HCC. Clinical trials are needed to establish the safety profiles and adverse effects, as well as to provide evidence-based data of the efficacy of the novel microalgae-based therapeutics against HCC. Continued research focused on clinical translation is therefore critical for realizing the potential of microalgae as novel interventions against HCC.

## Figures and Tables

**Table 1:** Microalgae-derived anticancer agents.

Compound	Source Microalgae	Primary Anticancer Mechanisms	Cancer Models Studied	References
**Carotenoids**				
Fucoxanthin	*Chaetoceros calcitrans*, *Phaeodactylum tricornutum*, *Nitzschia* sp., *Skeletonema marinoi*	Apoptosis induction, cell-cycle arrest, inhibition of proliferation, migration, invasion, endothelial cell tube formation	Liver, prostate, glioblastoma, colon	[71,72,73,74,75,76,77,78,79,80]
Astaxanthin	*Haematococcus pluvialis*, *Haematococcus lacustris*	Apoptosis induction, cell-cycle arrest, reduced proliferation, chemosensitization	Colon, lung	[81,82,83,84,85,86,87,88,89,90,91,92,93]
β-Carotene	*Dunaliella salina*	Apoptosis induction, cytotoxicity, antiproliferative effects, cell-cycle arrest	Lung, skin	[91,94,95,96]
Lutein	*Chlorella*	Cytotoxicity	Colon	[91,97]
Zeaxanthin	*Dunaliella salina*, *Porphyridium Purpureum*	Apoptosis induction, antioxidant and antiproliferative effects, cytotoxicity	Breast, lung, melanoma	[91,97,98,99]
**PUFAs**				
EPA	Several sp.	Antiproliferative effects, apoptosis induction	Several cancer cell lines	[100,101,102,103]
DHA	*Crypthecodinium cohnii*	Apoptosis induction, cell-cycle arrest, antiproliferative effect	Breast	[104,105,106]
**Polysaccharides**				
GA3P	*Gymnodinium* sp. A_3_	Inhibition of DNA topoisomerases I and II	Breast, glioblastoma, colon, lung, melanoma, ovary, renal, gastric	[107]
Exopolysaccharides	*Porphyridium cruentum*, *Chlorella* sp., *Nitzschia palea*, *Arthrospira platensis*, *Tetraselmis suecica*, *Thraustochytriidae* sp.	Cytotoxicity, apoptosis induction, antiproliferative effects	Lung, cervical, liver, breast, ovarian, colon, leukemia	[108,109,110,111,112,113,114,115,116]

**Table 2:** Biological effects of microalgae in murine models of HCC.

Murine Species	Induction of HCC	Microalgae/Cyanobacteria Species and Dosing of Animals	Significant Findings	Mechanistic Insights	References
Male albino rats (*Rattus rattus*	Dibutyl nitrosamine (DBN) precursors in drinking water for 6 months	*Spirulina platensis* (*SP*) For DBN + SP-treated group, rats were fed on a standard diet mixed with 1% SP powder for 12 months.	SP treatment reduced liver tumor incidence to 20% in the DBN + SP group as compared to 80% in the control DBN group, suggesting that SP could prevent DBN-initiated tumor development in the rat liver.	1. Cell-cycle inhibition 2. Induction of apoptosis. Potential pathways are p53/p21/Rb and p53/Bax/Bcl-2, respectively	[128]
Male Wistar rats	Choline-deficient diet supplemented with 0.1% ethionine in drinking water (CDE)	*Chlorella vulgaris* (CV) Rats were administered CDE + CV at 50, 150 and 300 mg/kg. Animals euthanized at 0, 4, 8, and 12 weeks.	Hepatoprotective effect of CV as evidenced by a significantly lower expression of liver tumor markers AFP (alpha-fetoprotein), TGF-β, M_2_-PK and OV-6 as compared to control animals.	1. Scavenging ROS 2. Enhancing antiproliferative effect 3. Induction of apoptosis	[131]
Male CD1 albino mice (*Mus musculus*)	Single intraperitoneal (i.p) injection of diethylnitrosamine (100 mg/kg), followed by 22 weekly i.p injections of carbon tetrachloride (0.5 mg/kg)	*Spirulina* sp. HCC-induced mice were administered *Spirulina* (at doses of 250 and 500 mg/kg body weight, respectively) for 4 weeks, beginning from week 25 until week 28 of HCC induction.	Considerable regression of tumors with decreased number of hepatic nodules and reduction in expression of AFP tumor marker, together with an increased survival rate.	1. Restoring antioxidant capacity and reducing oxidative stress 2. Inducing apoptosis by activating pro-apoptotic p53 and Bax, and suppressing anti-apoptotic Bcl-2. 3. Reducing tumor angiogenesis by inhibition of the angiogenic marker VEGF	[132]
Male Sprague Dawley (SD) rats	Rat hepatoma N1-S1 cells were implanted into liver lobes of SD rats under ultrasound guidance	*Coelastrella* sp. *Coelastrella* sp. F50 extract (300 mg/kg/day) was administered for 17 days.	Oral administration of the F50 extract impaired the progression of Novikoff hepatoma in experimental rats with significantly smaller tumors than those in the control group. Moreover, it was observed that expression of Ki-67 proliferative index was significantly reduced in F50-treated hepatoma tissues, together with increased apoptotic TUNEL (Terminal deoxynucleotidyl transferase dUTP nick end labeling) staining and downregulation of hepatic CSC markers CD133/ABCG2, with reduction in elevated serum aminotransferase/alanine transferase.	Inhibition of hepatic CSCs by blockade of cyclooxygenase-2/prostaglandin E2 axis	[130]
Male Wistar albino rats	Single i.p dose of carbon tetrachloride (CCl_4_) (0.5 mL/kg body weight)	*Anabaena oryzae* Gastric lavage of phycocyanin (isolated from *Anabaena oryzae*) at doses of 25, 50, and 100 mg/kg body weight/day for 14 days.	*Anabaena oryzae* phycocyanin when administered to CCl_4_-injured rats could mitigate CCl_4_-induced liver structural alterations induced by alleviating oxidative stress.	Antioxidant capacity	[133]
Male/ Female BALB/c mice	Subcutaneous injections of 1 × 10^5^ H22 murine hepatoma cells near the axilla	*Spirulina platensis* (*SP*) Fifteen days post-administration of H22 cells, mice were randomly divided into six groups: control group, SP phycocyanin—treated group, laser alone treated group, Selenium-enriched (Se)-phycocyanin treatment group given 0.2 mL Se-phycocyanin (10 mg/mL), phycocyanin PDT and Se-phycocyanin PDT treatment groups	Se-phycocyanin PDT group showed the strongest anticancer effect with 75.4% tumor inhibition rate followed by SE-phycocyanin (55.2%) and phycocyanin PDT group (52.6%). Glutathione peroxidase enzyme activity of Se-phycocyanin with or without laser treatment were higher than the phycocyanin PDT and control groups.	Antioxidant capacity	[134]
Female C3H/HeN and C3H/HeJ mice	Intradermal injection of 1 × 10^6^ MH134 murine HCC cells on the backs of C3H/HeN or C3H/HeJ mice	*Spirulina pacifica* Lipopolysaccharide (LPS) prepared from *Spirulina*, *E. coli* LPS, or saline was injected intraperitoneally on days 6, 13 and 20.	Administration of different doses of *Spirulina* LPS by injection suppressed tumor growth in C3H/HeN (which harbor the wild type toll-like receptor 4 (TLR4) gene) but not in C3H/HeJ mice (which has the mutated TLR4 gene). Serum levels of IL-17 and IL-23 decreased, whereas IFN-γ production by T cells increased in tumor-bearing C3H/HeN mice.	Because IL17/IL23 and IFN-γ levels are altered, it would appear that *Spirulina* LPS suppressed tumor growth by modifying the cytokine milieu in the tumor-bearing mice through the TLR4 pathway	[135]

## Data Availability

The original contributions presented in this study are included in the article. Further inquiries can be directed to the corresponding author.

## Abbreviations

The following abbreviations are used in the manuscript:

AFP	Alpha-fetoprotein
AgNPs	Silver nanoparticles
ALA	Alpha-linolenic acid
ALE	Adaptive laboratory evolution
BCLC	Barcelona Clinic of Liver Cancer
CAP	Cyanobacterium aponinum polysaccharide
CCl_4_	Carbon tetrachloride
cTACE	Conventional transarterial chemoembolization
CDE	Choline-deficient diet supplemented with ethionine
CSC	Cancer stem cell
CV	*Chlorella vulgaris*
DBN	Dibutyl nitrosamine
DEB-TACE	Drug-eluting bead transarterial chemoembolization
DHA	Docosahexaenoic acid
DOX	Doxorubicin
DOX-SP	Doxorubicin-loaded Spirulina embolic agent
DPPH	1,1-Diphenyl-2-picryl-hydrazyl
EMT	Epithelial–mesenchymal transition
EPA	Eicosapentaenoic acid
GMP	Good Manufacturing Practice
HCC	Hepatocellular carcinoma
IC_50_	Half maximal inhibitory concentration
ICI	Immune checkpoint inhibitor
IRE	Irreversible electroporation
i.p.	Intraperitoneal
LPS	Lipopolysaccharide
MAPK	Mitogen-activated protein kinase
MWA	Microwave ablation
PDA	Polydopamine
PDA-CV@PI	Polydopamine-coated *Chlorella vulgaris* loaded with PD-1 inhibitor
PDT	Photodynamic therapy
PI	PD-1 inhibitor
PUFAs	Polyunsaturated fatty acids
RFA	Radiofrequency ablation
ROS	Reactive oxygen species
RTKs	Receptor tyrosine kinases
SD	Sprague Dawley
Se	Selenium-enriched
SP	*Spirulina platensis*
TAE	Transarterial embolization
TACE	Transarterial chemoembolization
TARE	Transarterial radioembolization
TLR4	Toll-like Receptor 4
TKI	Tyrosine kinase inhibitor
TUNEL	Terminal deoxynucleotidyl transferase dUTP nick end labeling
